# Revision of the planthopper genus *Nycheuma* Fennah (Hemiptera, Fulgoromorpha, Delphacidae)

**DOI:** 10.3897/zookeys.462.6657

**Published:** 2014-12-10

**Authors:** Xiao-Hui Hou, Xiang-Sheng Chen

**Affiliations:** 1 The Special Key Laboratory for Development and Utilization of Insect Resources / College of Animal Sciences, Guizhou University, Guiyang, 550025, P. R. China; 2 Zunyi Medical University, Zunyi, 563099, P. R. China

**Keywords:** Hemiptera, Fulgoroidea, Delphacini, *Nycheuma*, synonymy, new record, taxonomy

## Abstract

Chinese species in the genus *Nycheuma* Fennah, 1964a (Hemiptera: Fulgoromorpha: Delphacidae: Delphacinae: Delphacini) are revised to include three species: *Nycheuma cognatum* (Muir, 1917), *Nycheuma dimorpha* (Matsumura, 1910) and *Nycheuma nilotica* Linnavuori, 1973. *Nycheuma coctum* Yang, 1989 is placed in synonymy with *Nycheuma nilotica* Linnavuori, 1973. *Nycheuma dimorpha* (Matsumura, 1910) is newly recorded from China. The generic characteristics are redefined. The main morphological characters, male genitalia of 3 species are described or redescribed and illustrated. A key to Chinese species in the genus is provided.

## Introduction

The delphacid genus *Nycheuma* was erected by Fennah (1964a) with *Dicranotropis capensis* Muir, 1926 as its type species. It belongs to the tribe Delphacini within subfamily Delphacinae (Hemiptera: Fulgoroidea: Delphacidae) (Fennah 1964a; Kuoh et al. 1983; Yang 1989; Ding 2006) and is easily separated from other members in this tribe by the following diagnostic features. Head including eyes slightly wider than pronotum. Vertex quadrate, wider at base than long submedially about 1.2:1. Frons median carina forked about level of ocelli. Antennae surpassing the level of frontoclypeal suture. Pronotum lateral carinae not reaching hind margin. Pygofer ventral margin with 3 small medioventral processes (*Nycheuma endymion* only 1 and *Nycheuma menuis* absent). Aedeagus with a long retrose process at apex (Kuoh et al. 1983; Yang 1989; Ding 2006). This genus is known to occur in the Afrotropical, Indo-Malayan, Australian and Pacific regions. To date, 9 species have been recorded in the worldwide, *Nycheuma afrocognata* Asche (Ivory Coast), *Nycheuma cognatum* (Muir) (Australia, China, Fiji, Philippines, Sri Lanka, West Caroline Is., Bonin Is., New Caledonia), *Nycheuma coronata* Asche (Ivory Coast), *Nycheuma dimorpha* (Matsumura) (Australia, Cape Verde, Italy, Ivory Coast, Nigeria, South Africa), *Nycheuma endymion* (Fennah) (Senegal), *Nycheuma menia* Fennah (Sudan), *Nycheuma menuis* Fennah (Sudan), *Nycheuma nilotica* Linnavuori (Sudan), *Nycheuma sectator* (Fennah) (Cameroon, Sudan: Blue Nile, Umm Banein) (Matsumura 1910; Muir 1917; Fennah 1958, 1963, 1964a, 1964b, 1969; Linnavuori 1973; Asche 1988). But only 2 species have been described in China. Only the host plant of *Nycheuma cognatum* is known (*Paspalum orbiculare* G. Forst), most of species in the genus *Nycheuma* feed unknown.

Here, the Chinese species of the genus *Nycheuma* are revised to include three species: *Nycheuma cognatum* (Muir, 1917), *Nycheuma dimorpha* (Matsumura, 1910) and *Nycheuma nilotica* Linnavuori, 1973. *Nycheuma coctum* Yang, 1989 is placed in synonymy with *Nycheuma nilotica* Linnavuori, 1973. *Nycheuma dimorpha* (Matsumura, 1910) collected from Datian National Natural Reserve, Hainan Province, is newly recorded from China. The generic characteristics are redefined. The main morphological characters and male genitalia of 3 species are described or redescribed and illustrated. A key for identifying the Chinese species of *Nycheuma* is also provided.

## Material and methods

The methods and morphological terminology used in this study follow that of Yang and Yang (1986) and Ding (2006). The genital segments of the examined specimens were macerated in 10% KOH and drawn from preparations in glycerin jelly using a light microscope. Illustrations of the specimens were made by using Leica MZ 12.5 stereomicroscope and enhanced using Adobe Photoshop 7.0 (Adobe Systems). Spinal formula means the numbers of apical spines of the hind tibiae and 1^st^ and 2^nd^ hind tarsomeres. The type specimens and materials examined are deposited in the Institute of Entomology, Guizhou University, Guiyang, Guizhou Province, China (IEGU).

## Taxonomy

### Nycheuma

Taxon classificationAnimaliaHemipteraDelphacidae

Fennah, 1964

Figs 1 –36

Nycheuma Fennah, 1964a: 145; Kuoh et al. 1983: 81; Yang 1989: 95; Ding 2006: 247.

#### Type species.

*Dicranotropis capensis* Muir, 1926, by original designation.

#### Description.

The characters used by Fennah (1964a), Kuoh et al. (1983), Yang (1989) and Ding (2006) are modified as follows:

**Body size.** Macropterous form, body length (including forewing): male 3.20–3.44 mm, female 3.70–4.32 mm.

**Coloration.** General color light yellowish brown to yellowish brown. Pronotum and mesonotum with carinae and border pale yellowish brown. The terminal of first segment and the base of second segment antennae dark brown (Figs 2, 14, 26). Metapleura with round spot dark brown. Abdomen brown to dark brown. Forewings hyaline, veins dark brown (Figs 3, 15, 27). Hindwings hyaline with veins dark brown.

**Figures 1–12.**
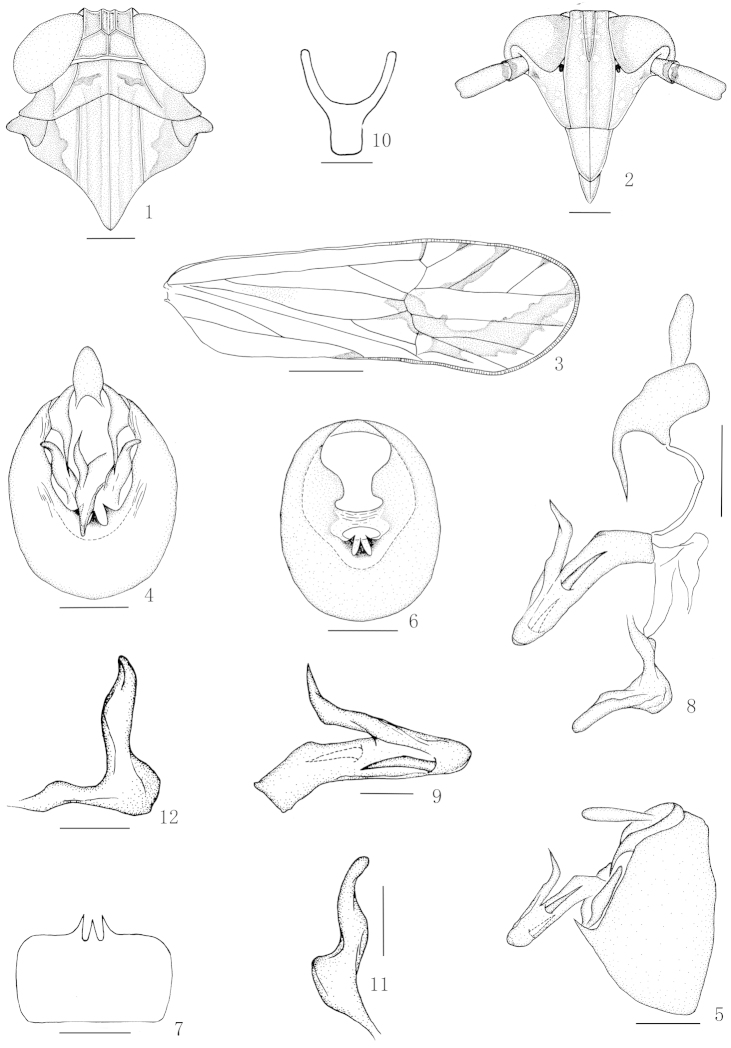
*Nycheuma cognatum* (Muir) **1** Head and thorax, dorsal view **2** Frons and clypeus **3** Forewing **4** Male genitalia, posterior view **5** Male genitalia, lateral view **6** Diaphragm of pygofer **7** Pygofer, ventral view **8** Anal segment, aedeagus, connective and genital styles, lateral view **9** Aedeagus, lateral view **10** Suspensorium **11** Genital style, posterior view 12 Right genital style, lateral view. Scale 1 mm (Figure **3**); 0.2 mm (Figures **1, 2, 4–8**); 0.1 mm (Figures **9–12**).

**Figures 13–24.**
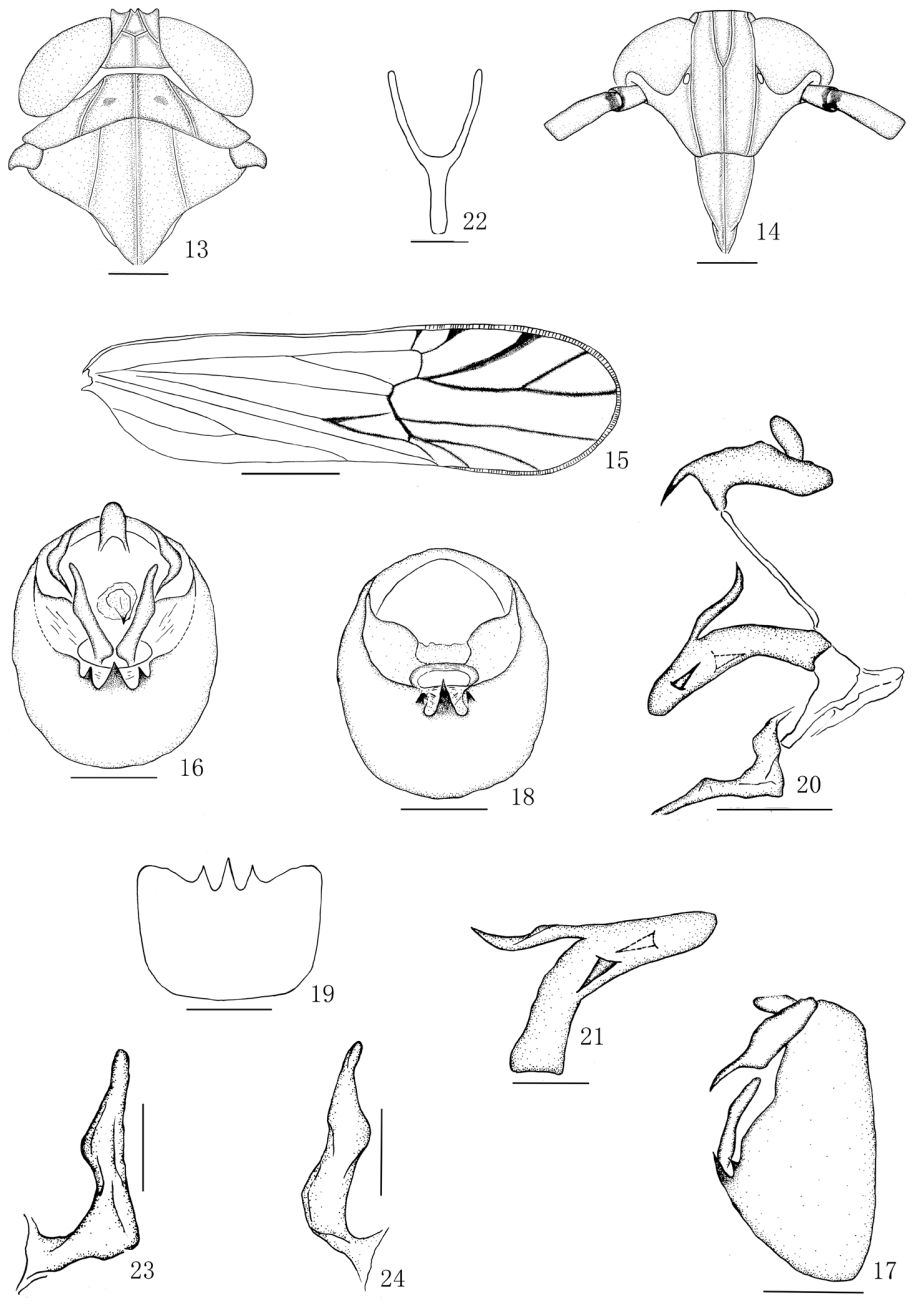
*Nycheuma dimorpha* (Matsumura) **13** Head and thorax, dorsal view **14** Frons and clypeus **15** Forewing **16** Male genitalia, posterior view **17** Male genitalia, lateral view **18** Diaphragm of pygofer **19** Pygofer, ventral view **20** Anal segment, aedeagus, connective and genital styles, lateral view **21** Aedeagus, lateral view **22** Suspensorium **23** Genital style, posterior view **24** Right genital style, lateral view. Scale 0.2 mm (Figures **13–20**); 0.1 mm (Figures **21–24**).

**Figures 25–36.**
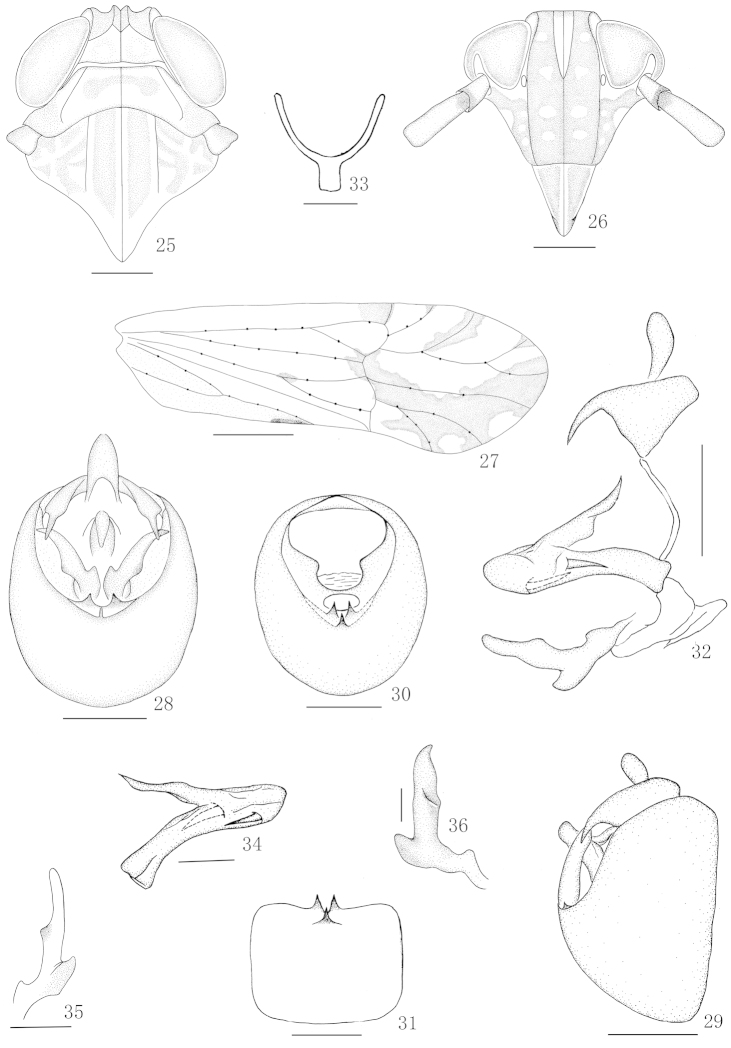
*Nycheuma nilotica* Linnavuori **25** Head and thorax, dorsal view **26** Frons and clypeus **27** Forewing **28** Male genitalia, posterior view **29** Male genitalia, lateral view **30** Diaphragm of pygofer **31** Pygofer, ventral view **32** Anal segment, aedeagus, connective and genital styles, lateral view **33** Aedeagus, lateral view **34** Suspensorium **35** Right genital style, posterior view **36** Left genital style, lateral view. Scale 0.5 mm (Figure **27**); 0.2 mm (Figures **25, 26, 28–32**); 0.1 mm (Figures **33–36**).

**Head and thorax.** Head, including eyes (Figs 1, 13, 25), as wide as pronotum or slightly wider. Vertex quadrate, shorter submedially than wider at base about 1:1.2, moderately rounding into frons, apical margin transverse with submedian carinae moderately prominent, Y-shaped carina feeble, submedian carinae not uniting at apex, basal compartment of vertex wider at base than greatest length about 2.2:1, than medium length about 2.7:1. Frons (Figs 2, 14, 26) in midline longer than wide at widest part about 2.0:1, widest at level of ocelli, lateral margins straight and converging distad beyond this level, median carina forked at base. Postclypeus in profile apical part of median carina bend. Rostrum with apical segment about as long as subapical. Antennae cylindrical, reaching slightly beyond frontoclypeal suture, basal segment longer than wide about 2:1, shorter than second about 1:2 (Figs 2, 14, 26). Pronotum (Figs 1, 13, 25) with lateral carinae not attaining hind margin. Spinal formula of hind leg 5–7-4. Posttibial spur with about 20 teeth.

**Male genitalia.** Pygofer short dorsally, long and strongly convex ventrally (Figs 5, 17, 29), posterior opening about as long as wide, laterodorsal angle not produced, lateral margins rather feeble, medioventral processes present (3 or 1 small processes) or absent (Figs 7, 19, 31). Diaphragm deeply impressed with dorsal margin membranous (Figs 6, 18, 30). Phallus rather long, laterally compressed, with a long retrose process at apex (Figs 8, 9, 20, 21, 32, 33). Suspensorium in posterior view Y-shaped (Figs 10, 22, 34). Genital styles simple, rather narrow, tapering distally, rectangulately or subacutely bent dorsad, if produced caudad at point of flexure, then lobe narrow and very small, divergent in opposite direction apically (Figs 11, 12, 23, 24, 35, 36). Anal segment of male (Figs 4, 5, 8, 16, 17, 20, 28, 29, 32) short, lateroapical angles widely separated, each produced ventrad in a spinose process.

#### Host plant.

*Paspalum orbiculare* Forst (Ding 2006).

#### Distribution.

Afrotropical, Indomalayan, Australian and Pacific regions.

#### Remarks.

In the genera of Delphacini, this genus is most similar to *Euidopsis* Ribaut, 1948 (with the single species *Euidopsis truncata* Ribaut, 1948), but differs in the following: Frons median carina forked level of ocelli (in *Euidopsis*, frons median carina forked above level of ocelli); antennae reaching the level of frontoclypeal suture (in *Euidopsis*, antennae reaching the level of end part of post-clypeus); metatarsal tibial spur with 20 small teeth on lateral margin (in *Euidopsis*, metatarsal tibial spur with 30 small teeth on lateral margin); pygofer ventral margin with medioventral processes (3 or 1) or absent (in *Euidopsis*, pygofer ventral margin with 1 small medioventral process); diaphragm without armature (in *Euidopsis*, diaphragm with 1 armature); aedeagus with 1 long retrose process arising near apex (in *Euidopsis*, aedeagus with 2 long retrose processes arising near apex).

#### Key to known Chinese species of *Nycheuma*

**Table:** 

1	Forewing of macropterous male without marking apically; phallus with process on right side near apex (Figs 3, 8)	***Nycheuma cognatum***
–	Forewing of macropterous male with marking apically; phallus with process on right side near middle (Figs 15, 20, 27, 32)	**2**
2	Pygofer ventral margin with 3 identical medioventral process, all processes in a row (Fig. 31); phallus with processes strong and long (Fig. 33); genital styles inner basal angle moderate and stout (Figs 35, 36)	***Nycheuma nilotica***
–	Pygofer ventral margin with 3 distinct medioventral process, intermediate process shorter than lateral processes, processes not in a row (Fig. 19); phallus with processes slender and short; genital styles inner basal angle obvious and fig-like (Figs 23, 24)	***Nycheuma dimorpha***

### Nycheuma cognatum

Taxon classificationAnimaliaHemipteraDelphacidae

(Muir, 1917)

Figs 1–12

Dicranotropis cognata Muir, 1917: 317.
Nycheuma cognatum (Muir), comb. by Fennah 1964a: 145; see also Fennah 1969: 37; Fennah 1971: 571; Fennah 1973–75: 89; Kuoh et al. 1983: 81; Ding 2006: 249.

#### Description.

Body length including forewing 3.44 mm (male), 3.95 mm (female).

**Coloration.** General color uniformly brown. Forewing subhyaline, brown, in brachypterous male with a large black marking at apex.

**Head and thorax.** Vertex (Fig. 1) shorter submedially than wide at base about 1:1.1, Y-shaped carina moderately distinct, basal compartment of vertex wider at base than greatest length about 1.8:1. Frons (Fig. 2) in midline longer than wide at widest part about 2.2:1, widest at level of ocelli. Postclypeus wider at base than frons at apex, slightly wider at base than length in middle line. Rostrum reaching to metatrochanters, apical segment distinctly shorter than subapical. Antennae (Fig. 2) surpassing level of middle of postclypeus, basal segment longer than wide about 1.7:1, shorter than second about 1:1.8. Post-tibial spur with about 27 teeth.

**Male genitalia.** Anal segment of male (Figs 4, 5, 8) moderately long, collar-shaped, lateroapical angles very widely separated, each produced caudad and slightly mesad in a stout spinose process. Pygofer in profile (Fig. 5) wider ventrally than dorsally, posterior margin strongly produced caudad medially, in posterior view (Figs 4, 6) with opening small, distinctly wider than long, lateral margin weakly defined, ventral margin shallowly concave, with 3 distinct medioventral processes, middle the longest. Phallus (Figs 8, 9) long, tubular, slightly arched upward medially, reflected cephalad at apex in a flagellum on right side, top of flagellum slightly turned mesad than laterad, pointed at apex, with a large, stout process at middle left and a smaller one near apical fourth right. Orifice terminal dorsad. Suspensorium (Fig. 10) Y-shaped, arms longer than stem. Diaphragm (Fig. 6) rather broad, membranous, triangularly incised dorsally. Opening for genital styles elongate oval. Genital styles (Figs 11, 12) slender, widely divergent, narrowing to apex, inner margin nearly straight, outer margin moderately produced laterad medially.

#### Material examined.

2 ♂♂, CHINA: Jianfengling National Natural Reserve (18°43'N, 108°53'E), Hainan Province, 17–20 Apr. 2009, collected by X.-H. Hou; 3 ♂♂, CHINA: Bawangling National Natural Reserve (19°05'N, 109°07'E), Hainan Province, 24–28 Apr. 2009, collected by X.-H. Hou; 1 ♂, CHINA: Datian National Natural Reserve (19°06'N, 108°47'E), Hainan Province, 21–23 Apr. 2009, collected by X.-H. Hou; 1 ♂, CHINA: Volcano Park (19°55'N, 110°13'E), Hainan Province, 6–8 Apr. 2009, collected by X.-H. Hou; 1 ♂, 1 ♀, CHINA: Datian National Natural Reserve (19°06'N, 108°47'E), Hainan Province, 9 Jul. 2007, collected by Z.-G. Zhang.

#### Host plant.

*Paspalum orbiculare* Forst.

#### Distribution.

China (Hainan, Taiwan), Philippines, West Caroline Is., Bonin Is., Sri Lanka, New Caledonia, Fiji, Australia.

### Nycheuma dimorpha

Taxon classificationAnimaliaHemipteraDelphacidae

(Matsumura, 1910)

Figs 13–24

Dicranotropis dimorpha Matsumura, 1910: 37.
Nycheuma dimorpha (Matsumura), comb. by Asch 1988: 195.

#### Description.

Body length including forewing 3.20–3.36 mm (male), 4.20–4.32 mm (female).

**Coloration.** General color dirty yellowish brown to yellowish brown. Vertex, pronotum and mesonotum (Fig. 13) dirty yellowish. Frons and genae (Fig. 14) dirty yellowish brown, with several circular spots light yellowish brown. Clypeus (Fig. 14) dark yellowish brown. Eyes blackish brown, ocelli reddish brown. Antennae general dirty yellowish brown, with apex of scape ring with dark yellowish brown. Thorax with ventral parts light yellowish brown to yellowish brown. Legs with trochanters light yellowish brown to yellowish brown. Forewings (Fig. 15) hyaline, with apex of irregular spot brown. Abdomen with ventral parts dirty yellowish brown.

**Head and thorax.** Head including eyes wider than pronotum about 1.1:1. Vertex (Fig. 13) wider at base than long submedially about 1.4:1. Pronotum (Fig. 13) slightly shorter than vertex submedially about 0.8:1. Mesonotum (Fig. 13) longer than pronotum and vertex combined about 1.5:1. Frons (Fig. 14) longer in middle line than wide at widest part about 2.1:1, widest about level of ocelli, lateral carinae nearly straight below ocelli, median carina forked at level of ocelli. Postclypeus (Fig. 14) wider slightly at base than frons at apex, as long as wide at base. Rostrum reaching metacoxae, apical segment distinctly shorter than subapical. Antennae (Fig. 14) reaching frontoclypeal suture, basal segment longer than wide about 1.6:1, shorter than second about 1:2.1. Post-tibial spur with about 26 teeth.

**Male genitalia.** Anal segment of male (Figs 16, 17, 20) short, moderately, collar-like, lateroapical angles separated and produced into a stout spinose process. Pygofer in profile (Fig. 17) distinctly longer ventrally than dorsally, with laterodorsal angle not produced, in posterior view (Figs 16, 18) with opening as long as wide, lateral margin weakly defined, ventral margin concave, with 3 small medioventral processes, in ventral view pointed at different level. Phallus (Figs 20, 21) large, tubular, apex reflected cephalad at right, turned dorsad near apex, with 2 long, stout processes directed basad, one on apical fifth left, another on apical third right. Suspensorium (Fig. 22) Y-shaped, arms distinctly longer than stem. Diaphragm (Fig. 18) narrow, partly membranous, dorsal margin distinctly concave. Opening for genital styles small, dorsal and ventral margins evenly planus. Genital styles (Figs 23, 24) moderately long, divergent, slender, wider at base than at apex, inner base angle obvious, inner margin planus, outer margin slightly sinuate, with a nodule-like process nearly middle.

#### Material examined.

3♂♂, 2 ♀♀, CHINA: Datian National Natural Reserve (19°06'N, 108°47'E), Hainan Province, 12–15 Apr. 2009, collected by X.-H. Hou.

#### Host plant.

Unknown.

#### Distribution.

China (Hainan: Datian).

### Nycheuma nilotica

Taxon classificationAnimaliaHemipteraDelphacidae

Linnavuori, 1973

Figs 25–36

Nycheuma nilotica Linnavuori, 1973: 105.
Nycheuma coctum Yang, syn. by Yang 1989: 98; see also Ding 2006: 247.

#### Description.

Body length including forewing 3.28–3.37 mm (male), 3.88 mm (female).

**Coloration.** General color pale yellowish brown. Apex of first antennal segment and base of second, abdomen and pygofer brown, metapleura with large rounded brown spot. Forewing (Fig. 27) hyaline, with brown marking on hind margin near end of clavus, anterior area of Cu, on ends of Sc_1_, Sc_2_, wider on R_1_, narrower on R_s_, oblique area along posteroapical area, reaching to end of M_1_ except for 3 hyaline areas.

**Head and thorax.** Vertex (Fig. 25) wider at base than long submedially about 1.1:1, at apex as wide as at base, basal compartment at base wider than greatest length about 1.8:1. Frons (Fig. 26) in midline longer than wide at widest part about 2.3:1, widest about level of ocelli, lateral carinae nearly straight below ocelli, median carina forked at level of ocelli. Postclypeus (Fig. 26) wider at base than frons at apex, slightly longer than wide at base. Rostrum reaching metacoxae, apical segment shorter than subapical. Antennae (Fig. 26) reaching frontoclypeal suture, basal segment longer than wide about 1.8:1, shorter than second about 1:2.0. Post-tibial spur with about 26 teeth.

**Male genitalia.** Anal segment of male (Figs 28, 29, 32) short, collar-shaped, lateroapical angles each produced into a long spinose process, widely separated each other, directed ventrad. Pygofer in profile (Fig. 29) distinctly longer ventrally than dorsally, in posterior view (Figs 28, 30) with opening longer than wide, lateral margins defined, ventral margin shallowly concave with 3 medioventral processes, in ventral view pointed at same level. Phallus (Figs 32, 33) large, tubular, apex reflected cephalad at right, turned dorsad near apex, with 2 long, stout processes directed basad, one on apical fourth left, another near middle right. Suspensorium (Fig. 34) Y-shaped, arms slightly longer than stem. Diaphragm (Fig. 30) not distinctly membranous, dorsal margin slightly concave. Opening for genital styles small, dorsal and ventral margins evenly convex. Genital styles (Figs 35, 36) moderately long, divergent, apical half slightly twisted, turned caudad apically, inner margin slightly sinuate, outer margin shallowly concave at apical half, outer angle pointed.

#### Material examined.

3 ♂♂, 3 ♀♀, CHINA: Datian National Natural Reserve (19°06'N, 108°47'E), Hainan Province, 10 Jul. 2007, collected by Q.-Z. Song and B. Zhang; 2 ♂♂, CHINA: Sanzhao Yangguangzui (22°16'N, 113°34'E), Guangdong Province, 8 Oct. 2008, collected by X.-H. Hou; 1 ♂, CHINA: Diaoluoshan National Natural Reserve (18°40'N, 109°52'E), Hainan Province, 14 Jul. 2008, collected by H.-R. Li.

#### Host plant.

Unknown.

#### Distribution.

China (Guangdong, Hainan, Taiwan).

## Supplementary Material

XML Treatment for Nycheuma

XML Treatment for Nycheuma cognatum

XML Treatment for Nycheuma dimorpha

XML Treatment for Nycheuma nilotica
